# A Comparative Study Between Conventional Sutures, Staples, and Adhesive Glue for Clean Elective Surgical Skin Closure

**DOI:** 10.7759/cureus.31196

**Published:** 2022-11-07

**Authors:** Kiran Mastud, Yashwant Lamture, Tushar Nagtode, Venkatesh Rewale

**Affiliations:** 1 General Surgery, Jawaharlal Nehru Medical College, Wardha, IND; 2 General Surgery, Jawaharal Nehru Medical College, Wardha, IND

**Keywords:** wound size, comparative study, adhesive skin glue, surgical staple, non-absorbable skin-sutures

## Abstract

As long as medicine has subsisted, wound closure has existed. When assessing a surgical wound, physicians are more than ever confronted with various sutures and other closure materials. The surgeon must choose the most appropriate material for that specific closure because not one substance is perfect in all circumstances. The history of suturing wounds is intertwined with the history of surgery. Wound suturing is a critical component of wound therapy, including suturing materials and techniques.

Suturing has been practiced for thousands of years. Although suture materials and techniques have changed, the goals like closing dead space, supporting and strengthening wounds until healing increases their tensile strength, approximating skin edges for an aesthetically pleasing and functional result, and reducing the risk of bleeding and infection remain the same.

Traditional skin closure with sutures requires time and effort, creates an unsightly scar, and requires infection removal. In this modern era, patients find it appealing when the operated location has a decent cosmetic appearance. The quicker, more comfortable, and most aesthetically pleasing method of skin closure is what surgeons are searching for. Assessing whether newer methods are superior to sutures and staples is necessary. Better cosmesis, flexibility, water resistance, painless sealed skin closure, and ease of application are all advantages of 2-octyl cyanoacrylate.

This article aims to provide an overview of the critical characteristics of traditional sutures, common suture materials, sticky glue, and staples, as well as additional closure materials.

## Introduction and background

"Scar" is a surgeon's signature [1]. Tissue approximation is a fundamental requirement for skin closure. A surgeon should aim for a successful tissue reunion and a functional and aesthetically pleasing scar. Wound closure techniques have progressed from the earliest innovations in suturing materials to more sophisticated tools like skin staplers, skin glue, and sticky tapes. Based on the effectiveness of modern suturing techniques, patients may experience fewer postoperative pain and wound infections, better cosmetic results, and shorter hospital stays.

The history of suturing extends back to the Masai of East Africa would insert acacia thorns around the edges of a laceration before plaiting plant fiber to connect them and close the wound [2]. When Dr. Harry Coover, a scientist at Kodak, first created cyanoacrylates in 1942, the current generation of skin adhesives began. The cyanoacrylates gained popularity in 1958 when Dr. Coover began publicizing them as powerful, quick-drying glue. 

Several combatants carried this powerful adhesive skin glue for rapid mechanical repairs on the battlefield during the Vietnam War (1955-1975). When warriors were hurt, blood and necessity always beckoned. It was found that cyanoacrylate glue, which polymerized and solidified when opened up to moisture, had a remarkable capacity to keep wounds closed. Field doctors eventually began applying it to wounds before transferring patients to army hospitals, and as a result, many lives were saved [1].

**Figure 1:**
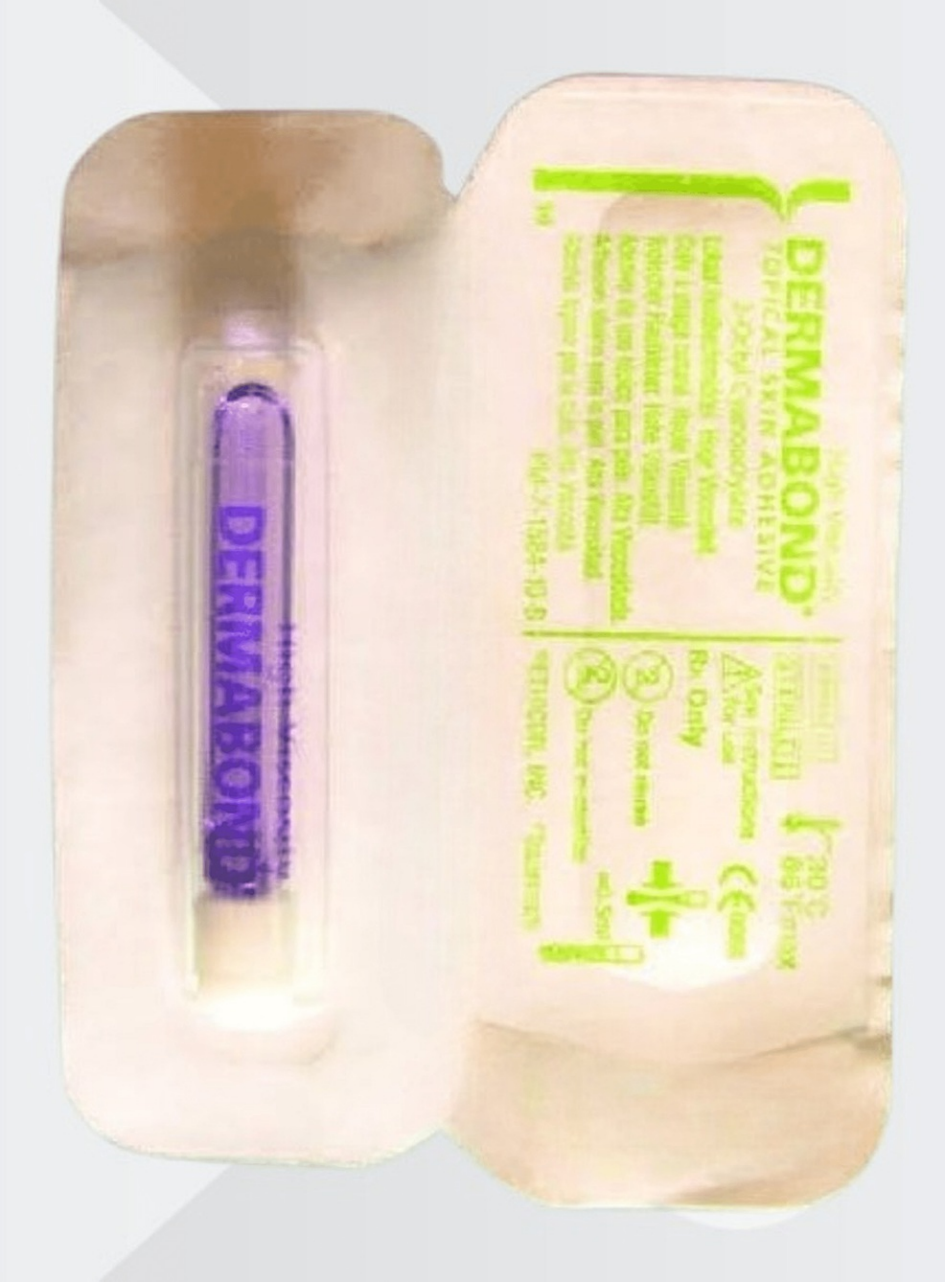
Skin adhesive glue (octyl cyanoacrylate)

The glue didn't take long to receive the United States Food and Drug Administration certification for allopathic use. Octyl-2-cyanoacrylate is presently utilized globally in hospitals, primarily for minor superficial wound healing and where the use of sutures would be inconvenient or difficult (Figure 1).

A liquid monomer called Dermabond glue (Ethicon Inc., Somerville, NJ) creates a solid tissue bond with a protective layer that strengthens it and prevents bacteria growth. Glue inhibits gram-positive (methicillin-resistant Staphylococcus aureus and S epidermidis) and gram-negative (Escherichia coli) microorganisms, according to an in vitro study. The glue also has the potential benefits of quick application and repair times. Within a year of the repair, it has been demonstrated to produce results that are cosmetically comparable to those of staples. Additionally, it was shown that patients accepted glue well.

The cyanoacrylates first rose to fame in 1958 when they were marketed as a potent, quick-drying glue. Alkyl cyanoacrylates are now utilized to make the most common tissue adhesives. After surgery, the method of wound closure has long been up for discussion. The capacity to know what to use, when to use it, and for how long makes up the core competency of a modern surgeon today. Each of the aforementioned skin closure methods is unique and has advantages and disadvantages of its own.

The best method to treat a specific wound is still debatable. The consequences of choosing the incorrect closure method for a particular wound might be severe. To compare skin suturing, skin staples, and sticky skin glue, it is necessary to investigate numerous elements of various types of skin closure techniques. 

## Review

Excellent material for wound closure should be non-allergenic, simple to make and use, and affordable. Any method of skin approximation must retain the skin edges in apposition for a sufficient time to allow healing. There are two types of sutures: absorbable and non-absorbable. Silk and ethilon are non-absorbable sutures. To increase its tensile strength, silk is braided. It knots and handles well. Although reliable yet conventional percutaneous interrupted silk sutures are prone to numerous drawbacks, they are still the gold standard for skin wound closure. On tissue implantation, silk tends to inflate, and the spaces between its braiding often become infiltrated with bacterial detritus and tissue that is actively growing. Ethilon is a synthetic monofilament suture material with a good memory that is black and not too tricky to handle. One must use numerous knots because the security of the first one is minimal. 

Stapler: It is quicker, more dependable, and manageable. Even wound tension is produced by the staple's constant depth and regular shape. 

Synthetic adhesives without solvents are cyanoacrylates. When applied to a surface, the monomer liquid initiates polymerization, forming a solid polymer layer. It offers a waterproof and antibacterial coating. A small layer is put over the entire wound, and the creation of a bond causes heat to be produced over the skin. 

The skin is then treated with cyanoacrylate tissue glues after the edges of the wound have been roughly trimmed. The liquid preparation polymerizes and creates a barrier impervious to water and germs, holding the margins of the wound together and enabling epithelialization. With time, cyanoacrylate glues deteriorate and shed, requiring no more appointments for removal.

Three groups make up a cyanoacrylate monomer's chemical structure: ethylene, alkyl, and cyano. Different cyanoacrylate compounds have other chemical, physical, and mechanical properties, and these variations are related to the alkyl group's hydrocarbon side chain. The rate of polymerization, rate of degradation, toxicity, flexibility, and other properties of the glue in its polymerized state are all controlled by this variable area.

Early research found that short alkyl chain cyanoacrylate monomers exhibited higher levels of reactivity and hence had faster rates of polymerization and breakdown. This has significant effects on how short-chain cyanoacrylates are used. Early animal experiments showed that, despite quick skin closure, the polymerization process is highly exothermic because quickly polymerizing monomers reach higher temperatures and are more likely to cause thermal tissue injury. Formaldehyde and alkyl cyanoacetate breakdown products are toxic and can cause severe phytotoxicity and inflammation in rat models, although they weren't carcinogenic. Because of this, therapeutic application as a biological tissue adhesive is not currently appropriate for short-chain cyanoacrylate monomers like ethyl- and methyl-2-cyanoacrylate [3]. 

Butyl- and octyl cyanoacrylates are the two types of cyanoacrylate currently on the market. The octyl-based cyanoacrylates have historically been assumed to be stronger and more flexible than the shorter butyl cyanoacrylates due to the considerable length of their side chain. Numerous earlier experiments have repeatedly demonstrated that octyl cyanoacrylate is more durable and pliable than any butyl cyanoacrylate now on the market [3].

Incision Length

The present study's illustrations show that the incision was 0 to 10 cm long. Therefore, skin glue was only used to close small to medium surgical wounds. This relates once more to the previously mentioned limitation of cyanoacrylates (Dermabond), which is that they cannot be used to close extensive skin wounds. Numerous researchers only tested this material in certain types of damage, such as the episiotomy wounds that Adoni et al. closed using tissue adhesive [4]. In skin incisions from head and neck surgery, this substance was examined by Samuel et al. and Maw et al. [5,6]. Another study by Simon et al. showed that cyanoacrylate is a favored technique for the cutaneous closure of lacerations directed away from Langer's lines [7]. As a result, no study investigated using adhesive material to close lengthy cuts. 

Time is taken for skin closure

In a study by Ridgway et al., the average time taken for closure of celiotomy incision in neck surgeries with glue was substantially more than with skin staplers, with a mean difference of 67 seconds [8]. The average time for skin closure in the adhesive group of arthroplasty patients was reported to be 100 seconds, compared to an average of 30 seconds for the placement of staples total hip replacement (THR) vs total knee replacement (TKR) [9]. To close scalp incisions from neurosurgical procedures, Chibbaro et al. found no discernible difference between surgical adhesive glue and skin staples [10].

The current study demonstrates that staples' wound closure time was quicker than glue and sutures.

Post-operative pain

The patients themselves used the visual analog scale to rate their post-operative discomfort. According to measurements taken at twelve hours, twenty-four hours, forty-eight hours, three days, and seven days, the glue group experienced much less post-operative discomfort than the group that used staples, followed by the group that used skin sutures. Comparable studies support the findings of this investigation by Gaertner et al. and Singh et al., which demonstrated an association between abdominal wounds healed with sutures and higher postoperative pain [11,12].

Previous research by Zempsky et al. and Arunachalam et al. evaluated postoperative pain using a visual analog scale and found that sticky glue closures resulted in less post-operative pain. Still, these investigations failed to reach statistical significance [13,14]. Our study demonstrated solid statistical significance, demonstrating that glue users experienced less post-operative discomfort than those who received sutures or staples.

Complications/ASEPSIS score 

In the three groups in the current study, erythema, purulent exudates, serous exudates, and wound gaping were all visible. In contrast to staples and sutures, only erythema had a statistically meaningful comparison, which explains why there were fewer incidences of erythema in the glue population. The correct glue application method is also essential, as the improper way can lead to wound dehiscence.

In their studies, Khan et al. and Chibbaro et al. found no discernible differences between the two groups' data collection methods [15,16]. As opposed to the glue group, cases in the staple group experienced gaping. The meta-analysis, which included data from four well-known trials, discovered a significant difference in the proportion of wounds with dehiscence, favoring suture closure with no sign of heterogeneity [17]. However, Blondeel et al. found that the new tissue adhesive formulation provides epidermal wound closure equivalent to commercially available devices with a trend toward decreased incidence of wound infection in a series of 209 patients treated with octyl-2- cyanoacrylate and commercially available devices after the closure of long surgical incisions [18].

The current study also notably showed that the ASEPSIS score in the population of glue users was lower than that of the other two groups, indicating that glue had lower infection rates than staples and sutures. On day three, this had a significant "p" value. Day five gave out signs of importance.

Cosmesis of wounds

These three groups of patients were analyzed for the wound's cosmetic result using the Modified Hollander and visual analog scale (VAS) cosmesis measures on the seventh post-operative day, the first month, and the third month. On the seventh post-operative day, the average cosmesis score for all three groups was numerically in favor of glue. Still, this difference could not be shown to be statistically significant. After a month, there was a numerical difference in the mean cosmesis scores across the three research groups, which statistically proved that the patients with skin glue had better cosmesis than the other two. 

In a randomized series involving 43 patients who underwent groin incision surgeries, Keng et al. discovered that the glued wounds consistently received higher cosmetic scores (mean score of 4.71 at four weeks) than the subcuticular wounds (mean score of 4.00 at four weeks), with a P value of 0.05 [19]. The results of the current investigation, which were highly significant, demonstrated that glue had greater cosmesis than sutures and staples.

According to the material costs, sutures were the most economically advantageous of the three skin closure techniques. The price increased when the skin was closed, subcuticular with polyglactin sutures. The adhesive group in our study experienced a substantially shorter hospital stay than the other groups, and this difference had great statistical significance. According to Jones et al., the use of skin adhesive (20.3 Euros) was generally found to be significantly more cost-effective than the use of sutures (29.3 Euros) (p 0.001) [20]. The authors conclude that there was little difference in results between the closure techniques but that glue was superior to sutures economically.

An economic analysis of the expense of using either 4-0 monocryl/vicryl or cyanoacrylate glue to close laparoscopic incisions was done in a study from Texas, U.S.A. [21]. The time saved during surgery resulted in a mean cost savings of 303 dollars per patient in the cyanoacrylate group. These articles lend credence to our investigation, which found that adhesive was significantly more cost-effective than staples and sutures.

## Conclusions

According to the results of the current prospective comparison study between adhesive skin glue, skin staples, and sutures, the glue group appears to have superior post-operative pain tolerance, fewer wound complications, shorter hospital stays following surgery, and better cosmetic results overall. However, there was a lengthier application period than staples and a little greater cost than with sutures. An appealing alternative to using sutures for superficial skin closure is the idea of a surgical tissue adhesive. Using sticky glue speeds up skin closure and reduces the length of the surgical procedure. It produces a flexible, water-resistant skin closure and improves the appearance.

Overall, because of its additional virtue of being bacteriostatic and the lack of a post-operative removal necessity, skin glue surpasses to be the closure method of choice for the desired wound. Therefore, it can be said that 2-Octylcyanoacrylate is safe to employ in clean elective surgery skin closure.
